# Occupational Tuberculosis in Denmark through 21 Years Analysed by Nationwide Genotyping

**DOI:** 10.1371/journal.pone.0153668

**Published:** 2016-04-15

**Authors:** Mathias Klok Pedersen, Aase Bengaard Andersen, Peter Henrik Andersen, Erik Svensson, Sidse Graff Jensen, Troels Lillebaek

**Affiliations:** 1 International Reference Laboratory of Mycobacteriology, Statens Serum Institut, Copenhagen, Denmark; 2 Department of Infectious Diseases, Copenhagen University Hospital, Copenhagen, Denmark; 3 Department of Infectious Disease Epidemiology, Statens Serum Institut, Copenhagen, Denmark; 4 Department of Pulmonary Medicine, Gentofte Hospital, Hellerup, Denmark; Fundació Institut d’Investigació en Ciències de la Salut Germans Trias i Pujol, Universitat Autònoma de Barcelona, SPAIN

## Abstract

Tuberculosis (TB) is a well-known occupational hazard. Based on more than two decades (1992–2012) of centralized nationwide genotyping of all *Mycobacterium tuberculosis* culture-positive TB patients in Denmark, we compared *M*. *tuberculosis* genotypes from all cases notified as presumed occupational (N = 130) with *M*. *tuberculosis* genotypes from all TB cases present in the country (N = 7,127). From 1992 through 2006, the IS6110 Restriction Fragment Length Polymorphism (RFLP) method was used for genotyping, whereas from 2005 to present, the 24-locus-based Mycobacterial Interspersed Repetitive Unit-Variable Number of Tandem Repeat (MIRU-VNTR) was used. An occupational TB case was classified as clustered if the genotype was 100% identical to at least one other genotype. Subsequently, based on genotype, time period, smear positivity, geography, susceptibility pattern, and any reported epidemiological links between the occupational cases and any potential source cases, the occupational case was categorized as confirmed, likely, possible or unlikely occupationally infected. Among the 130 notified presumed occupational cases, 12 (9.2%) could be classified as confirmed and 46 (35.4%) as unlikely, accounting for nearly half of all cases (44.6%). The remaining 72 cases (55.4%) were categorized as possible. Within this group, 15 cases (11.5%) were assessed to be likely occupational. Our study shows that genotyping can serve as an important tool for disentangle occupational TB in high-income low incidence settings, but still needs to be combined with good epidemiological linkage information.

## Introduction

It is well documented that tuberculosis (TB) may constitute an occupational hazard [1]. Traditionally, the conversion of the tuberculin skin test and contact investigations have been the only method to connect an occupational TB case with a potential source case [2]. During the last two decades, DNA genotyping techniques applied on *Mycobacterium tuberculosis* have greatly increased our understanding of *M*. *tuberculosis* transmission. From the 1990s and onwards, sporadic cases of occupational TB were documented using genotyping techniques [3–7]. Still, only few studies, mainly focusing on health care workers (HCWs), have examined occupationally transmitted TB using genotyping techniques on a larger scale [8–11].

Denmark is a low TB-burden country with an overall incidence of 6.3 per 100,000 (2013) and most cases are found in specific ethnic or socially marginalized risk groups in the population [12]. Occupational cases are reported through the mandatory national notification system whenever a physician presumes a TB case to be occupational (5–10 cases each year). However, the true magnitude of occupational transmission is unknown, as no systematic analysis has been done before.

As all culture-verified *M*. *tuberculosis* cases have been genotyped on a nationwide basis in Denmark since 1992, it is possible to analyse occupational TB transmission by molecular means. The underlying assumption is that active TB will bring the vast majority of potential source cases in contact with the health system, and given the nationwide centralized diagnostics, *M*. *tuberculosis* genotyping for more than two decades and the mandatory notification system, these sources cases will be uncovered. Thus, any source case to any presumed occupational case with a clustered *M*. *tuberculosis* isolate should ideally exist in the central database enabling us to possible confirm or reject occupational transmission events. In this way, by comparing genotypes, we evaluated all *M*. *tuberculosis* culture-positive cases notified with presumed occupational TB in Denmark through 21 years.

## Materials and Methods

### Study design, databases and genotyping

This was a retrospective nationwide register study based on centralized *M*. *tuberculosis* genotyping from 1992 through 2012.

In Denmark, TB is a notifiable disease as part of national surveillance. The notification form has the option to indicate a case as *presumed occupational*. This indication is based solely on the attending physician’s judgement of the case history with no formal evidence required. Epidemiological linkage information on each case may be indicated on the notification sheet; however, this information is not systematically given. Case information, such as age, address, work place, country of presumed transmission and epidemiological linkage information when available, are stored at the Department of Infectious Disease Epidemiology at Statens Serum Institut (SSI), which serve as the national institute for surveillance and control of infectious diseases in Denmark. Also at SSI, the International Reference Laboratory of Mycobacteriology (IRLM) performs all cultures, culture-based drug susceptibility testing and molecular typing of *M*. *tuberculosis* strains in Denmark. Since 1992, more than 94% of all culture-verified new and recurrent *M*. *tuberculosis* cases diagnosed have been genotyped. The results have been stored in a laboratory register at the IRLM together with the laboratory findings and case-related information. From 1992 through 2006, the IS*6110* Restriction Fragment Length Polymorphism (RFLP) method was used as genotyping tool [13]. The IRLM is now using the 24-locus-based Mycobacterial Interspersed Repetitive Unit-Variable Number of Tandem Repeat (MIRU-VNTR) method [14,15] and have tested all *M*. *tuberculosis* cases since 2005, i.e. there is a two-year overlap with the RFLP testing [16]. The two methods have comparable discriminatory power [14].

### Cohort and analysis

We compared the strain genotyping results from all *M*. *tuberculosis* culture-positive cases notified as presumed occupational with all genotyped *M*. *tuberculosis* culture-positive cases in Denmark from 1992 through 2012. Additional epidemiological information on occupation and whereabouts of the index case and potential source cases at suspected time and places of transmission were included in the analysis when available. A TB case was classified as clustered if the RFLP or MIRU-VNTR type of the isolate was 100% identical to the type of at least one other case in Denmark during the study period. Based on genotype, epidemiological information, cluster size, time period, infectiousness (pulmonary TB and microscopy positive smear grading), geography, and susceptibility pattern of the potential source and index cases, the index case was categorized as *confirmed*, *possible* or *unlikely* occupational. A *confirmed* case was defined as a culture-positive TB case clustering with a suspected source case with a direct epidemiological link between the two cases (Table 1). A *possible* case was defined as a culture-positive TB case clustering with at least one other TB case, but without a known direct epidemiological link. Occupational cases with unique (non-clustered) isolates were also considered *possible* if information was available that the occupational transmission could have occurred outside Denmark, e.g. if a sailor with a unique isolate reported TB as occupational. An *unlikely* case was a case, which either did not cluster or was clustering with a confirmed non-occupational (community) epidemiological link case or solely with one or more potential source cases considered non-contagious due to extra pulmonary location of TB. To further assess the risk of occupational TB, a sub analysis was performed to define possible cases that were more likely. These sub groups of *likely* occupational cases were separated from the *possible* group based on stronger epidemiological links as well as on smaller cluster sizes. A *likely* case could e.g. be a health care worker with no known direct epidemiological links to potential source cases, but with an isolate belonging to a small cluster in which a few of the possible source cases were smear-positive and had been admitted to the hospital, where the index case worked.

**Table 1:** Definition of categories.

Category	Definition
*Confirmed*	A culture-positive TB case clustering with a potential source case with a direct epidemiological link between the two cases.
*Likely*	A culture-positive TB case with stronger potential epidemiological links than the possible category, however without a confirmed link. A stronger link was based on probable coincidence of geography and time interval with contagious potential source cases as well as on a smaller cluster size (<10).
*Possible*	A culture-positive TB case clustering with at least one other smear-positive TB case, but without a known direct epidemiological link or cases with unique genotypes and presumed infection abroad.
*Unlikely*	A culture-positive TB case which did not cluster or was clustering with a confirmed non-occupational (community) epidemiological link case or solely with one or more potential source cases considered non-contagious due to extra pulmonary location of TB.

### Statistics

The quantitative variables were described using median and range. The qualitative variables were described as numbers and percentages out of the total.

### Ethics

The necessary permissions to access and process data were obtained (Danish Data Protection Agency, J. no. 2012-54-0100). The study was a retrospective survey based on register data (not a clinical trial)—thus, according to Danish legislation, additional ethical approval and informed written consent from participants were not required. All analyses are presented anonymously.

## Results

**Fig 1:**
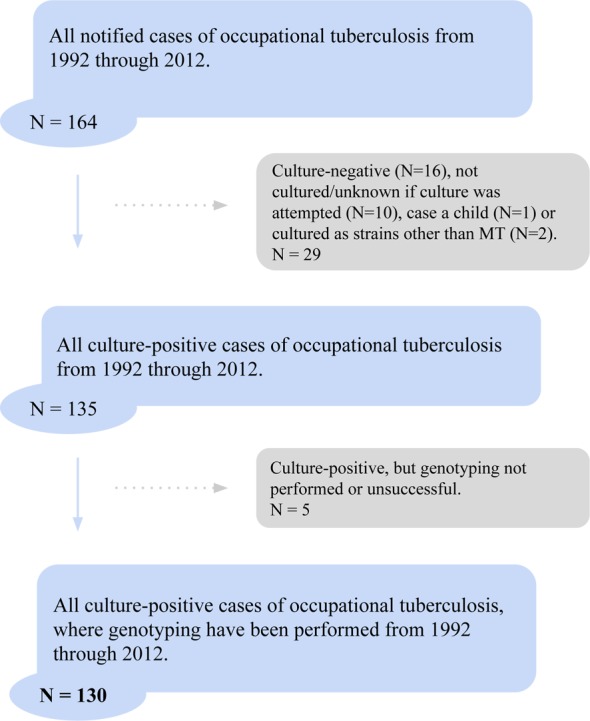
Inclusion flow-chart of presumed occupational TB cases from 1992 through 2012.

During the studied period, 164 cases were notified as *presumed occupational*, corresponding to a mean of nearly eight (7.8) per year (Fig 1). Of the 164 cases, 34 were excluded from the study. Among these, 16 were culture-negative, 10 were not cultured or it was unknown if culture had been attempted, 2 were not *M*. *tuberculosis*, 1 was a child, and 5 *M*. *tuberculosis* culture-positive cases were excluded as genotyping had not been done.

In total 130 TB cases from 1992 through 2012 were evaluated. They were all notified as presumed occupationally infected, *M*. *tuberculosis* culture-positive and genotyped with RFLP or MIRU-VNTR. Their genotyping information was compared to 7,127 genotyped cases in our database, corresponding to 78.6% of all notified TB cases (N = 9,069) and 94.6% of all culture-positive TB cases (N = 7,532) in the country.

Of the 130 cases included, 12 (9.2%) were *confirmed* as occupational, as their genotypes were 100% identical to the genotypes from reported epidemiological links with sputum smear-positive TB (Table 2). Occupational TB was *unlikely* for 46 cases (35.4%), as they were unique or as they clustered with extra pulmonary TB cases only or with a non-occupational (community) epidemiological link case. The remaining 72 cases (55.4%) were classified as *possible*, as clustered potential source cases were identified, but no clear epidemiological link was present. Of these, 15 cases (11.5%) were assessed to be *likely* occupational based on a stronger, although not directly confirmed, epidemiological link and based on a smaller cluster size, thereby leaving 57 (43.9%) as *possible* cases. No cases had notified epidemiological links to potential source cases that were not cultured or genotyped. Half of the *confirmed* cases (N = 6) belonged to clusters with more than 50 potential source cases, while the other half (N = 6) belonged to clusters with only a few potential source cases (less than 10).

**Table 2:** Classification of notified and culture-positive presumed occupational TB cases based on nationwide DNA fingerprinting of *M*. *tuberculosis* from 1992 through 2012. Data are presented as n (%) and median (range).

	All	Confirmed	Likely	Possible	Unlikely
Category	130 (100)	12 (9.2)	15 (11.5)	57 (43.9)	46 (35.4)
**Sex**					
Male	73 (56.2)	4 (33.3)	6 (40)	40 (70.2)	23 (50)
Female	57 (43.8)	8 (66.7)	9 (60)	17 (29.8)	23 (50)
**Age yrs**	44 (19–84)	45 (19–66)	47 (26–62)	43 (20–80)	45 (20–84)
**Site of TB**					
Pulmonary	117 (90)	12 (100)	14 (93.3)	50 (87.7)	41 (89.2)
Extrapulmonary	7 (5.4)	0 (0)	1 (6.7)	4 (7)	2 (4.3)
Both	6 (4.6)	0 (0)	0 (0)	3 (5.3)	3 (6.5)
**HIV positive**	2 (1.5)	0 (0)	0 (0)	1 (1.8)	1 (2.2)
**Country of origin**					
Denmark	100 (76.9)	12 (100)	11 (73.3)	47 (82.5)	30 (65.2)
Other	30 (23.1)	0 (0)	4 (26.7)	10 (17.5)	16 (34.8)
**Country of presumed infection**					
Denmark	103 (79.2)	12 (100)	15 (100)	39 (68.4)	37 (80.4)
Denmark or abroad	9 (6.9)	0 (0)	0 (0)	5 (8.8)	4 (8.7)
Abroad	18 (13.9)	0 (0)	0 (0)	13 (22.8)	5 (10.9)

**Fig 2:**
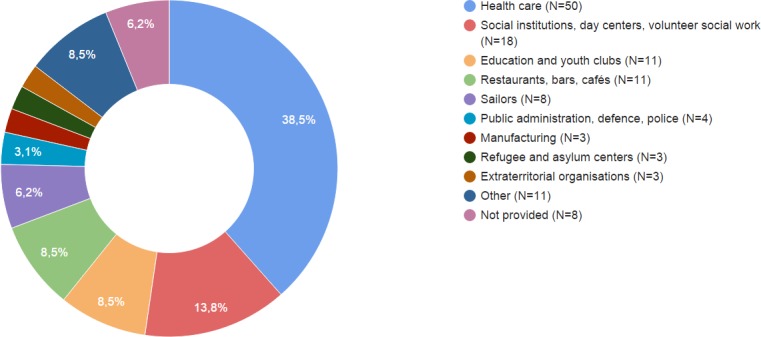
Distribution of notified presumed occupational cases based on industry (N = 130).

More than one third of the notified presumed occupational cases in the cohort (38.5%) were employed in the health sector, whereas 13.8% were social workers, typically in social institutions or as volunteers in social organizations or centers for alcohol and/or drug abusers (Fig 2). In the group of health care workers (HCWs), only six of 50 (12%) were directly *confirmed*, whereas eight (16%) were categorized as *likely*, 18 (36%) as *possible*, and another 18 (36%) as *unlikely* occupationally infected. The remaining six of the *confirmed* cases were occupied with social work (N = 3), in education (N = 1), in the armed forces (N = 1), and in a bar (N = 1).

In total, 56% were men and 44% were women. The median age was 44 years (range 19–84). All cases in the cohort were susceptible to the standard four-drug treatment regimen. Two cases were HIV positive. The reported country of presumed transmission was Denmark for 103 of the cases (79.2%) and abroad for 18 cases (13.9%), among which 13 cases were in the *possible* group, five cases in the *unlikely* group and none in the *likely* or *confirmed* group. For nine cases (6.9%), transmission in either Denmark or abroad was reported. Many of the cases (N = 55) lived in the Capital Region, whereas the rest were equally distributed in the rest of Denmark.

## Discussion

To our knowledge, this is the first nationwide study of occupational TB primarily based on *M*. *tuberculosis* genotyping techniques. By molecular means, spanning two decades, we demonstrated that nearly half (44.6%) of all 130 genotyped presumed occupational TB cases in Denmark could be fully clarified as *confirmed* (9.2%) or *unlikely* occupational (35.4%) when compared to a pool of 7,127 genotyped strains. The other half of the presumed occupational cases (55.4%), were in the more unclarified category *possibly* occupational. However, additionally 15 (11.5%) of the *possible* cases could be categorized as *likely* occupational.

More than one third (35.4%) of notified presumed occupational cases could be categorized as *unlikely*. Most cases in this group had unique genotype isolates. When these cases did cluster, it was with extra pulmonary TB cases or with a confirmed non-occupational (community) epidemiological link case. Less than one-tenth (9.2%) of the presumed occupational cases could be *confirmed*. Many index cases were part of larger clusters resulting in many potential source cases; if no clear epidemiological link was present, a direct transmission link could not be confirmed. In some instances, the notification form was lacking information on occupation and why occupational transmission was assumed. This explained why more than half (55.4%) of the cases could not initially be classified further than *possible*. Another reason for the lack of further classification of the *possible* cases was based on the fact that about a quarter of these cases were assumed to be infected abroad, hence genotypes for potential source cases were not available. When assessing the *possible* group in more detail, 15 cases could be classified further as *likely* occupational. These cases clustered with only few people from the same area of Denmark and they reported an occupation where contact with potential contagious source cases was considered to be possible, however, without a definite link. An in-depth epidemiological retrospective investigation could presumably lead to more clarification of the *likely* group although details might be very difficult to remember given the two decades time frame of the study.

HCWs constituted more than one third (38.5%) of the notified presumed occupational cases. In a metaanalysis by Menzies et al., HCWs were found to have a 2–3 times higher risk of TB morbidity [17]. Sepkowitz et al. found a relative risk of clustered vs. non-clustered TB for HCWs of 2.7, indicating a higher risk of recent transmission for HCWs [8]. In Hamburg, Germany, Diel et al. found that recent transmission of TB was strongly associated with health care work; for eight out of 10 HCWs in their database of 848 fingerprints, occupational infection could be confirmed based on clustering with a known epidemiological and work-related link [9]. In the Netherlands, de Vries et al. found that nearly every second case (42%) of TB in a HCW was caused by exposure in the workplace, but their category of confirmed cases was defined broader and thus not based solely on clustering with known occupational links [10]. Contrary to this, and also in the Netherlands, van Deutekom et al. found reduced risk for clustered cases in HCWs indicating a lower risk [11]. We *confirmed* six of 50 (12%) occupational cases among HCWs during two decades and assessed eight of 50 (16%) likely based on our more strict criteria, while 18 of 50 (36%), about one third, could be determined *unlikely*. Since about 175,000 persons work in the health care sector in Denmark (2012) this indicates a very limited risk of contracting TB in the health care sector compared to the general incidence of TB in Denmark of 6.3 per 100,000 per year. The apparent low level of occupational TB in Denmark may–aside from a general low TB incidence in the country—be explained by well-implemented TB prevention protocols [18,19], underscoring the need for these. However, the study was not designed for risk assessments; we only examined cases with active and culture-verified TB and did not assess the prevalence of latent infections.

Only sporadic information was available on the duration of exposure for the cases in our study. However, one of the confirmed cases of occupational TB transmission was seen after extremely short exposure; this case has previously been reported by Kamper-Jørgensen et al. [20]. This underscores the additional role genotyping has in contact tracing as conventional methods for this case would most likely have pointed on other more obvious potential links in which duration of exposure was longer.

There are some limitations to our study. We have overlapping RFLP and MIRU genotyping methods for the years 2005 and 2006 only. This could prevent us from finding potential source cases from before 2005 for occupational TB cases from 2007 and onwards. However, for all occupational cases from 2007 and onwards with epidemiological linkage to potential source cases from before 2005, comparable RFLP and MIRU-VNTR genotype information (translation key) was present for both the presumed occupational cases and the corresponding potential source cases. Thus, this should not affect our classification of confirmed cases significantly. In addition, we might overestimate the unlikely group, as unique occupational cases from 2007 and onwards theoretically could cluster with potential source cases before 2005. However, as we always had comparable genotypes to potential source cases at least two years backwards, and because most develop TB within the first two years after infection, this should not affect the unlikely group significantly either. In addition, only 10 cases from 2007 and onwards had unique MIRU-VNTR isolates with no RFLP typing done.

The underlying assumption in our study—that potential *M*. *tuberculosis* culture positive source case genotypes ideally should exist in our register—holds two limitations. First, we cannot rule out that transmission has occurred from source cases diagnosed outside Denmark or from source cases not diagnosed at all. However, in the majority of notified presumed occupational cases, transmission was expected to have occurred in Denmark and if transmission was in fact expected to have occurred outside Denmark, the cases were classified in the more indeterminate group *possible* (unless if these cases had better reasons to be categorized as unlikely, for instance if they were clustering with common strains in Denmark). Second, without any epidemiological information, clustered cases could just as well be community- rather than occupationally linked. Here we relied primarily on accessible, but often limited, epidemiological linkage information from the TB notification sheets.

The initial assessment of TB cases to be “presumed occupational” was done by the attending physician. Potentially this could have lead to an over reporting of presumed occupational cases (cases reported for the benefit of doubt) and thus to a relative overestimation of the proportion of unlikely cases in our study. However, this broad occupational notification criteria in Denmark allowed us to evaluate more potential occupational cases ideal for the purpose of the study.

The major strength of the study is the genotype based confirmation of nearly one tenth of presumed occupational cases and the disproving of more than one third of cases over the 21-year period. This could have implications for control measures at work, in particular if the occupational assessment is done routinely within a short time frame, so epidemiological information on possible modes of transmission is fresh in mind and can be collected and analysed. A timely analysis of genotyping data could also improve the applied contact tracing as presumed source cases in many instances can be disproved, thereby focusing the epidemiological investigation and resources at other potential (non-occupational) source cases. In addition, adding genotyping results to epidemiological linkage information could influence decisions on insurance claims.

DNA fingerprinting of *M*. *tuberculosis* isolates has provided a higher resolution of transmission events, as demonstrated for occupational related exposures in our study. However, RFLP and MIRU-VNTR are less effective in distinguishing occupational from community transmission events when only limited epidemiological information is present. New, potentially better, genotyping techniques are emerging. Especially, Next Generation Sequencing (NGS) might provide a higher resolution analysing transmission dynamics [21,22]. In the future, it is likely that NGS will provide even better information on occupational cases, especially cases such as the *likely* and *possible* occupational cases in our study.

## Conclusion

Our study demonstrates that genotyping can serve as an important supplement analysing occupational TB, but still, it does not obviates good epidemiological linkage information. Nearly half (44.6%) of all notified culture-positive presumed occupational TB cases from 1992 through 2012 could be clarified as either *confirmed* or *unlikely* based on genotyping of the *M*. *tuberculosis* strains in combination with the existing limited epidemiological linkage information. The rest (55.4%) could be defined as *possible* and among these, further 15 cases could be defined as *likely* occupationally infected. Future studies could focus on analysing occupational TB using NGS to gain an even higher resolution of potential transmission events.
